# Genome Instability at Common Fragile Sites: Searching for the Cause of Their Instability

**DOI:** 10.1155/2013/730714

**Published:** 2013-09-05

**Authors:** Annapaola Franchitto

**Affiliations:** Section of Molecular Epidemiology, Department of Environment and Primary Prevention, Istituto Superiore di Sanità, Viale Regina Elena, 299-00161 Rome, Italy

## Abstract

Common fragile sites (CFS) are heritable nonrandomly distributed loci on human chromosomes that exhibit an increased frequency of chromosomal breakage under conditions of replication stress. They are considered the preferential targets for high genomic instability from the earliest stages of human cancer development, and increased chromosome instability at these loci has been observed following replication stress in a subset of human genetic diseases. Despite their biological and medical relevance, the molecular basis of CFS fragility in vivo has not been fully elucidated. At present, different models have been proposed to explain how instability at CFS arises and multiple factors seem to contribute to their instability. However, all these models involve DNA replication and suggest that replication fork stalling along CFS during DNA synthesis is a very frequent event. Consistent with this, the maintenance of CFS stability relies on the ATR-dependent checkpoint, together with a number of proteins promoting the recovery of stalled replication forks. In this review, we discuss mainly the possible causes that threaten the integrity of CFS in the light of new findings, paying particular attention to the role of the S-phase checkpoint.

## 1. Introduction 

Genome instability is a common feature of cancer cells, and defects in DNA replication or in the replication checkpoint greatly increase this chromosome instability [1]. Common fragile sites (CFS) are regions of the human genome especially prone to breakage under condition of mild replication stress and are often found rearranged in cancer cells [2]. Chromosomal instability at these loci precedes the instability in the other genomic regions and is thought to be a driving force in cancer progression. The connection between CFS and cancer highlights the importance of the regulation of DNA replication to prevent cancer development. A direct involvement of CFS in cancer has not been yet established; however, a significant association between fragile sites and chromosome aberrations found in tumour cells has been demonstrated [3–6]. Chromosomal rearrangements often lead to alteration of gene products, which may acquire oncogenic potential or loss of tumour suppressor functions. Although the mechanisms of these processes are not completely clarified, it is likely that DNA breakage at CFS may represent an initiating event. Several studies have revealed that oncogene-induced replication stress preferentially targets fragile sites in premalignant cells, and it is becoming widely accepted that many if not all gross chromosomal rearrangements accumulating in solid tumours may originate at fragile sites [7–9]. 

DNA replication is a fundamental process to the life of a cell, but several challenges can threaten genome integrity by interfering with progression, stability, and proper resumption of replication after fork arrest [10]. Thus, inadequate handling of stalled forks or defects of DNA replication can lead to accumulation of mutations and genomic aberrations [10]. Accordingly, defective DNA replication is considered responsible for the majority of the chromosomal abnormalities arising in human tumours [11], and mutations in genes that protect genome integrity during DNA replication can cause a variety of human genetic diseases, such as Werner, Bloom, and Seckel syndromes as well as Fanconi anaemia, which lead to genome instability and predisposition to cancer [12]. 

Here, the current view of the origin of CFS fragility will be discussed in the light of studies indicating that instability at fragile sites may be the end result of problems encountered during DNA replication. 

## 2. CFS and DNA Replication

CFS are large genomic regions, spanning hundreds to thousands of kilobases that possess common features but show often different chromosome localizations in different cell types or tissues [13]. They are defined as specific chromosomal loci normally stable in cultured cells, which display gaps and breaks on metaphase chromosomes under certain conditions of replication perturbation. It is important to note that all the inducers of CFS can potentially stop elongation of DNA replication, thus only concentrations that partially inhibit replication without arresting the cell cycle can lead to CFS expression. The most typical inducer of a large part of CFS is aphidicolin, an inhibitor of the replicative DNA polymerases alfa, delta, and epsilon [14]. Until now, about 80 CFS have been identified, and their expression varies greatly among individuals, but two of them, FRA3B and FRA16D, are fragile in all of the individuals examined [15] and appear to be more prone to breakage in the human genome [14, 16]. Interestingly, sequences of FRA3B, the most active common fragile site in human lymphocytes, are late replicating [17]. Moreover, a previous study showed that replication along FRA3B was asynchronous during cell cycle and that breakage preferentially occurred on the chromosome with the late replicating allele, and thus it has been hypothesised that the inability to complete replication, in the presence of aphidicolin or some other cellular stress, predisposes to CFS instability [18]. Accordingly, treating cells with aphidicolin more than 10% of FRA3B sites is left unreplicated in G2 phase [17, 18]. 

Notably, a number of studies on replication timing have revealed that replication along FRA7H, FRA16D, FRA1H, and FRA2G starts correctly but then becomes delayed or prolonged, and aphidicolin exposure leads to a further slowdown of fork progression, so that fragile sites enter G2 phase unreplicated with very high frequency [17, 19–22].

Altogether these observations clearly indicate that a common feature of CFS is delayed replication, substantiating the hypothesis that CFS are regions intrinsically difficult to replicate. Moreover, these findings emphasize a close correlation between replication perturbation and the appearance of instability at CFS, as a relation of cause-effect. 

## 3. Potential Sources of CFS Instability 

**Figure 1:**
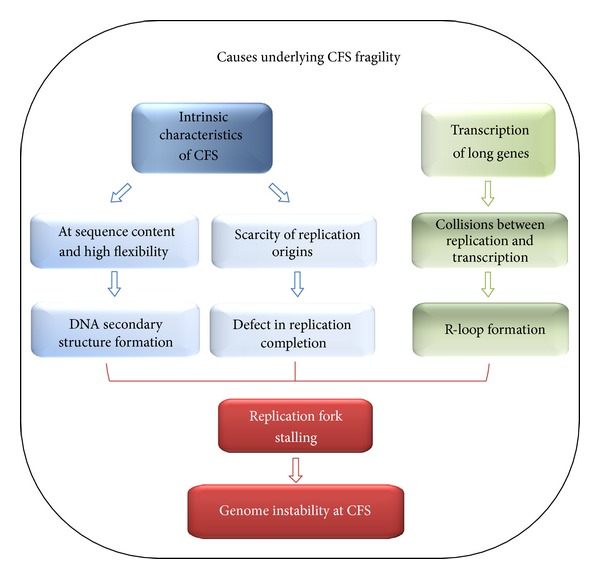
General scheme of the potential sources underlying CFS fragility and the final impact on genome stability.

Mounting evidence suggests that CFS instability stems from multiple factors, such as intrinsic characteristics of fragile regions and events that directly interfere with replication process (Figure 1). 

Computational analysis performed on a subset of fragile sequences has indicated that CFS contain frequent AT-rich islands [23], without any repeat motifs such as expanded trinucleotide or minisatellite repeats, which are responsible for fragility of another class of fragile loci, the rare fragile sites [24, 25]. These AT-rich sequences show a high flexibility, which confers them the ability to readily fold into secondary structures following the unwinding of the double DNA helix. Since these clusters are significantly more stable than random sequences with the same length and base composition [23], it is likely that their tendency to adopt such structures might confer proneness to fork stalling or perturbation of replication elongation. However, there is no evidence that such secondary structures actually form in vivo. Indeed, there is only indirect evidence that such sequences may perturb DNA replication because of their potential ability to adopt complex secondary structures. For instance, in yeast cells a polymorphic AT repeat that might form cruciform DNA structures within FRA6D determined replication fork stalling and increased chromosome breakage, mimicking what might happen at human CFS, also independently from replication stress [26]. Perhaps the most compelling, yet indirect, proof that intrinsic features of a CFS sequence are linked to breakage at those sites is provided by the observation that a stable ectopic integration of FRA3B into nonfragile loci recapitulates the CFS-like phenotype [27]. If formation of such cruciform structures does actually occur in vivo in human cells, thus replication polymerase pausing may occur, and long single-stranded DNA (ssDNA) regions produced at stalled forks may lead to ATR-dependent checkpoint activation, blocking the firing of new replication origins, preventing entry into mitosis, and promoting repair. Interestingly, the Werner helicase, which is implicated in the resolution of alternative DNA structures and CFS maintenance [28], is necessary to alleviate stalling at CFS maybe allowing bypass of unusual structures detrimental to DNA polymerase delta progression [29]. 

Apart from the possibility that CFS form secondary structures that may impair replication fork progression, novel findings strongly support a role for replication origin density in determining the fragility of CFS. Differences in the replication dynamics between origins mapped within the FRA3B versus nonfragile regions have been assessed [21]. Comparing the abundance of DNA nascent strand, a significant less newly replicated DNA at FRA3B than in the nonfragile regions has been detected. Furthermore, aphidicolin treatment did not increase the level of nascent DNA strand [21]. Although the assay used did not allow clearly defining the mechanism, the study suggests that low efficiency of origins located within CFS, with respect to those in nonfragile regions, may be responsible for replication perturbation along fragile sites. 

Significant advances in understanding the mechanism of fragile site instability have been recently achieved from analyses using the DNA-combing technique combined with fluorescent in situ hybridization (FISH). A fascinating new concept has been proposed by Debatisse's group to explain how these loci are so fragile [30]. The idea is that CFS expression is epigenetically defined. The study proposes that fragility of FRA3B in lymphoblastoid cells, but not in fibroblasts, is not due to fork slowing or stalling but to a scarcity of initiation events, which forces forks coming from flanking regions to cover long distances to finish replication. In addition, in the presence of DNA polymerase inhibitor, fork speed is impaired and replication at these loci risks to remain partial, making the unreplicated regions more prone to breakage [30]. Notably, Debatisse's studies have also demonstrated that commitment to fragile site instability depends on the same paucity of replication origins in different cell types, but that different chromosomal regions are committed in each cell type [31]. These observations completely substantiate the epigenetic nature of CFS expression, and emphasize the importance of replication origin density in the maintenance of CFS stability. Even though no sign of fork stalling along FRA3B has been detected, this could only mean that travelling forks only pause very transiently within the CFS, being the reason of pausing reverted quickly, making a direct assessment of this pausing hard because of technical constraints. 

Indeed a direct evidence for fork stalling at CFS has been provided [32]. Interestingly, most of the origins within the human FRA16C are already activated under normal growth conditions, clearly indicating that replication of this region is intrinsically perturbed [32]. Consistently, FRA16C region shows high levels of fork stalling compared to the whole genome, and this arrest preferentially occurs close to the AT-rich sequences. Under replication stress, the replication is further perturbed and more forks arrested at AT-rich sequences. However, CFS are unable to compensate for replication stress, and the inability to activate additional origins may impede the completion of replication leading to CFS destabilization. 

Recently, it has been proposed that also the transcription process may contribute to the fragility of CFS. Indeed, a number of CFS have been mapped to the coding regions of large human genes, and it has been well established that transcription of such genes requires long time to be completed, so that transcription and replication may occur at the same time. In this case, transcription machinery and replication forks may collide, resulting in R-loop formation, considered as a rare byproduct of transcription able to threat genome stability [33, 34]. In the presence of replication fork perturbation, DNA polymerase inhibits the elongating RNA polymerase and stable R-loops are created at the site of blockage, thereby contributing to breakage at long CFS-associated genes [34]. Interestingly, this instability can be suppressed by preventing R-loop formation, that is, through the enzymatic activity of RNase H1 that specifically degrades the RNA strand in RNA-DNA hybrids [34]. However, it is important to note that this mechanism cannot justify the fragility of all the CFS, as only about half of them are associated with large genes. Furthermore, recent findings suggest that replication forks travel similarly along CFS and genome-wide, leading to the conclusion that R-loop formation cannot be the prevalent way to affect fork movement [30]. 

Given that about one-hundred CFS have been recognised in human cells and as they clearly differ in sequence composition, the most biologically realistic explanation for their fragility is that all the molecular basis of the CFS instability hypothesised so far might be valid, perhaps each of them affecting specifically a subset of CFS at time. 

However, this apparent paradox should not prevent investigators to find some unifying model to CFS fragility. Indeed, all the mechanisms put forth up to now imply that progression of replication forks within CFS is perturbed. Secondary structures and replication/transcription interference might hamper fork progression directly while paucity of replication origins might force travelling forks to “spontaneously” collapse. Thus, it is likely that proteins involved in stabilization and safe recovery of the replication fork or in correctly engaging recombination at collapsed forks play an essential role in the maintenance of integrity at these loci independently to the downstream origin of the fragility. 

## 4. Replication Checkpoint Is Actively Involved in the Maintenance of Fragile Site Integrity

One of the most compelling evidence lines that replication is perturbed at CFS and that correct handling of perturbed forks is important for CFS stability is provided by the observation that genetic downregulation of ATR, the upstream S-phase checkpoint kinase [35, 36], dramatically and specifically, affects fragility of CFS, even in the absence of aphidicolin [37]. 

A detailed description of the ATR-signaling pathway is beyond the scope of this review and can be found in other reviews (i.e., [38]); however, it is important to mention that replication checkpoint signaling is stimulated by formation of extensive RPA-coated ssDNA regions, an intermediate produced at stalled forks, and involves subsequent recruitment and modification of various components of the signaling cascade. A large body of evidence has revealed that this complex pathway, once activated, regulates origin firing, cell cycle arrest, stabilization, and resumption of stalled forks [39–41]. A clear connection between replication checkpoint function and CFS was first established by seminal works from the group of Glover [37, 42]. The results of the studies demonstrated that ATR plays an important function in response to fork stalling arising at CFS. Indeed, ATR disruption or hypomorphic mutation, but not the related kinase ATM, greatly increases chromosome instability at CFS, both under unperturbed replication and treatment with aphidicolin. Although loss of ATM is not sufficient to induce CFS expression, however, ATM plays a role in CFS stability when ATR is lacking. Indeed, concomitant depletion of ATR and ATM leads to significant increase in CFS breakage as compared to ATR deficiency alone [32]. It is important to note that ATR preferentially interacts with FRA3B region and that its kinase activity is required for binding after aphidicolin treatment [43].

Several studies reported that multiple components of the ATR pathway such as CHK1 [44], HUS1 [45], and Claspin [46], or other ATR substrates, profoundly affected CFS integrity when defective. But how can replication checkpoint promote CFS stability, and most importantly, are the genetic determinants controlling replication checkpoint functions after genome-wide replication arrest conserved even when replication is perturbed specifically at CFS? 

Analysis of replication dynamics shows that aphidicolin treatment greatly slows the replication rate in proximity to CFS origins [21], suggesting that local perturbation of replication fork progression is sufficient to trigger extensive cell cycle arrest. From this point of view, the main checkpoint outcome, activation of the downstream CHK1 kinase, would be the critical point to preserve genome integrity. Indeed, CHK1 may suppress late replication origin firing and arrest cell cycle progression to provide cells the adequate time to resolve the problem [47]. Thus, the high CFS expression observed in checkpoint mutants might underlie the lack of proper CHK1 function after fork stalling at these loci and derive from S-M progression with CFS regions largely unreplicated. In addition to cell cycle regulation, CHK1 has been implicated also in maintaining replication fork integrity following replication inhibition and CHK1 inhibition or genetic downregulation leads to accumulation of DNA double strand breaks (DSBs) in S-phase cells [40, 48]. Stabilization of travelling forks could be another key function in maintenance of CFS integrity by the replication checkpoint, given that replication origins may be underrepresented in some CFS loci [30, 32]. Interestingly, and in agreement with the previous observation suggesting that loss of ATM alone does not influence CFS stability [37], all the checkpoint factors reported to control CFS stability so far, that is ATR, HUS1, and Claspin, also regulate CHK1 activation [49]. Downregulation of all these proteins, however, does not affect CFS expression similarly to what was reported in CHK1-depleted cells. Moreover, highest levels of CFS instability are observed only in ATR-defective cells, suggesting that the replication checkpoint actually regulates CFS integrity by multiple mechanisms and not just through CHK1-dependent cell cycle arrest or fork stabilization.

When DNA synthesis is perturbed, an important function of the replication checkpoint is to preserve the integrity of existing replication forks. In this context, the protective function of the replication checkpoint can be carried out by modulating the activity of some proteins involved in resolution of DNA secondary structures or processing replication intermediates, in order to avoid DNA breakage at forks. We recently reported that ATR-mediated regulation of the WRN RecQ helicase is sufficient to prevent DSBs at perturbed forks after replication stress induced genome-wide by hydroxyurea treatment [50, 51], suggesting that WRN is crucial for this replication checkpoint-mediated function. Interestingly, WRN and ATR act in a common pathway also to maintain chromosome integrity at CFS [28]. Even though WRN is a dual helicase/exonuclease enzyme, compelling evidence strongly supports a key role for WRN helicase activity in preserving CFS expression [28, 52]. Given the specific requirement of the WRN helicase activity for maintenance of CFS stability and the high propensity of CFS to adopt DNA secondary structures during DNA replication, it is likely that the helicase activity of WRN is instrumental to the unwinding of these structures in order to facilitate replication fork progression. This WRN function as fork protector could be regulated by the replication checkpoint preventing the disassembly of the replisome or providing enzymatic activities for the removal of certain DNA structures that stop fork movement. This is in agreement with the proposed coordinated action of WRN and DNA polymerase delta in the replication of DNA substrates containing G4 tetraplex structures [53]. Interestingly, WRN deficiency recapitulates ATR defects in terms of fragile site instability either upon aphidicolin treatment or under unperturbed conditions [28]. Hence, it is conceivable that the role of the WRN helicase activity could be coordinated with that of ATR in the replication checkpoint and that the well-described ATR-dependent stabilization of stalled forks may basically be carried out through phosphorylation and regulation of WRN by ATR. 

Finally, replication checkpoint could protect CFS integrity promoting the “repair” of unreplicated DNA regions in late S/G2 through the regulation of recombination-based postreplication repair mechanisms. Notably, it has been demonstrated that CHK1, independently of its role in stabilizing replication forks, may activate recombination through RAD51 phosphorylation [54]. This mechanism, however, would be expected to operate as back-up pathway whenever replication at CFS has been left incomplete because of inefficient activation of checkpoint in S-phase. Interestingly, we have recently reported that WRN-deficient cells accumulate ssDNA, as well as increased number of RAD51 foci, after aphidicolin treatment, and that depletion of RAD51 hyper-sensitizes WRN-deficient cells to aphidicolin [52]. Since RAD51 has been implicated in postreplication repair of ssDNA gaps accumulating behind the fork after replication perturbation [55], it is reasonable to assume that the elevated ssDNA accumulation and RAD51 foci formation observed in WS cells may reflect an extensive usage of RAD51-dependent postreplication gap repair at unreplicated CFS regions. Moreover, since RAD51 is involved in the maintenance of CFS stability in wild-type cells [56], lack of proper execution of RAD51-dependent postreplication repair may underlie chromosomal abnormalities at CFS. 

Therefore, the data reported so far clearly suggest that the pathway responding to replication fork stalling may function, perhaps at multiple levels, as an integral part of the mechanism regulating the integrity of the fragile genomic regions. 

## 5. Maintenance of CFS Stability during Mitosis

Recently, several lines of evidence indicate that maintenance of fragile site stability can be achieved also outside the S-phase and even after the G2-phase by pathways acting in mitosis [56–58]. 

Indeed, it has been reported that DSBs are formed at CFS, demonstrating the colocalization of *γ*H2AX, a marker for the induction of DSBs, with broken CFS in metaphase chromosomes [56]. Similarly, CFS were found colocalizing with RAD51 and phospho-DNA-PKcs foci, although it was not known whether DSBs derived from intermediates originated to bypass the DNA secondary structures or as a consequence of checkpoint escape [56].

However, since fragile sites are genomic regions where completion of replication may be difficult, it is conceivable that some cells can enter G2 with unreplicated DNA that can give rise to intertwined DNA structures that, if left unresolved, can degenerate into pathological structures. Indeed, these structures have been observed, and they were called ultrafine DNA bridges (UFBs). Those DNA filaments connecting two segregating chromosomes are thought to be the end result of recombination and/or replication intermediates not properly processed during S/G2-phase [57, 58]. In the last few years, a direct role for Fanconi anaemia (FA) pathway and RecQ helicase Bloom syndrome protein (BLM) has been demonstrated in the prevention and resolution of UFBs [57, 58]. FA proteins and BLM are thought to play a role in response to replication stress, and mutations in their genes have been associated with severe human diseases, the Fanconi anaemia and Bloom syndrome (BS), characterized by genome instability and cancer predisposition. A role for FA pathway in the regression of replication fork structures into a four-way junction has been reported in vitro, suggesting that stalled forks may represent the preferred in vivo substrate [59]. Interestingly, FA pathway is strongly activated in response to aphidicolin treatment and disruption of that pathway results in enhanced CFS expression [60]. BLM is implicated, in complex with RMI2, RMI1 and topoisomerase IIIa, in the resolution of converging replication forks and in the dissolution of double Holliday junction structures by means of topoisomerase IIIa decatenation activity. A concerted action of BLM and FA pathway on stalled forks during the S-phase has been reported [61–63]. Interestingly, more recently a cooperation of BLM and FA pathway during mitosis has been envisaged [57, 58]. In a previous study, the existence of BLM-associated UFBs was demonstrated [57]. These structures are not revealed by conventional DNA staining, but they are marked by BLM and FANCD2, colocalize with fragile regions, and represent sites of chromatid linkage, probably derived from unresolved replication intermediates [57, 58]. A model to explain how FA pathway and BLM collaborate after induction of mild replication stress has been provided [57, 58]. When replication is partially inhibited by aphidicolin and fork stalling occurs, FA pathway is necessary to stabilize the ssDNA catenanes formed at CFS throughout late S/G2 and probably into mitosis. In the absence of FANCD2 activity, these structures undergo breakage visible as gaps and breaks on metaphase chromosomes, resulting in the well-documented high expression of fragile sites in FA cells, whereas, in BS cells inefficient resolution of these catenanes results into anaphase UFBs accumulation. In case of failure or inappropriate resolution of UFBs, chromosomal breakage at CFS can occur, and micronuclei containing fragile site DNA can be detected leading to chromosomal instability. 

Even though FANC proteins and BLM have been placed respectively upstream and downstream RAD51-dependent recombination events, it is currently unknown whether they act in a common pathway also to protect chromosome integrity at CFS. Similarly, it is unknown if unresolved UFBs are then targeted by nucleases thought to resolve recombination intermediates. Interestingly, some resolvases are regulated through phosphorylation in mitosis [64], and, at least after oncogene-induced replication stress, MUS81 appears to be involved in the generation of chromosome breakage at CFS [65]. Clearly, more investigations are required to clarify these interesting points.

## 6. Conclusions

**Figure 2:**
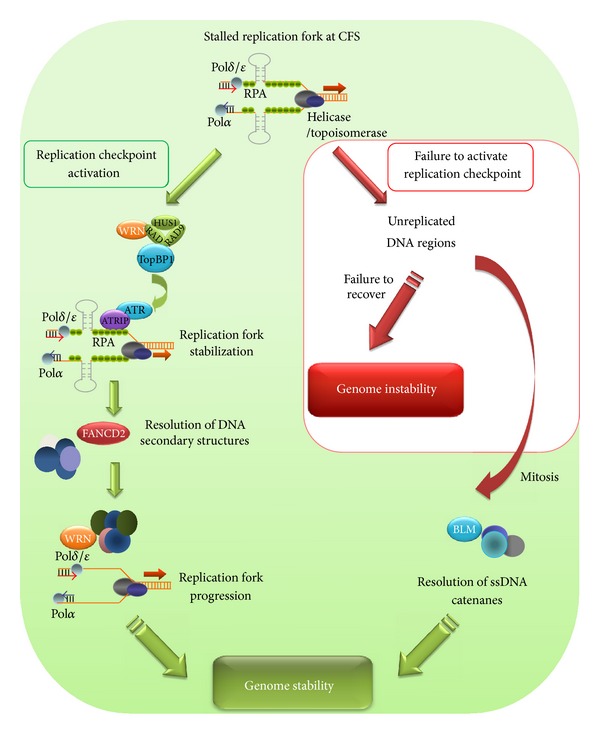
Schematic representation of the mechanisms involved in the maintenance of stability at common fragile sites. After replication, inhibition DNA secondary structures are formed within CFS leading to fork stalling. The replication checkpoint is triggered, and several proteins were recruited to recover stalled forks. Among the proteins involved in the safe resumption of replication forks are FANCD2 and WRN helicase. However, in case of failure checkpoint activation or in absence of key proteins, chromosomal abnormalities take place giving rise to genome instability (see text for details).

Taking into account all data concerning the possible mechanisms that govern the stability of fragile regions, it is possible to imagine a model which integrates all available information (Figure 2).

After partial inhibition of DNA replication, secondary structures could be adopted by CFS resulting in fork stalling. The ATR-dependent replication checkpoint is activated, and several proteins are recruited to deal with replication problems. Among the proteins, FANCD2 is necessary to stabilize stalled forks, and WRN helicase to resolve DNA secondary structures formed at CFS to allow the safe recovery of replication fork progression. However, in case of defective checkpoint activation or in the absence of FANCD2 or WRN function, unreplicated DNA regions are generated and ssDNA catenanes can be produced. These structures require BLM to be processed, and in the absence of this activity chromosomal abnormalities can arise resulting in genome instability. 

Although much progress has been made in understanding the underlying causes of common fragile site instability, a clear link between replication process and DNA breakage at these loci is still missing. A key role seems to be played by the ability of cells to stabilize stalled forks and assure their safe recovery. Otherwise, stalled forks could disrupt replication fork progression possibly resulting in the formation of large DNA unreplicated regions, which could pose a serious threat to genome stability. More detailed information on how cells defend themselves against this threat may come from a better elucidation of mechanisms by which proteins stabilize and/or recover stalled forks, avoiding degeneration into chromosomal instability.
